# Heart failure assessed based on plasma B-type natriuretic peptide (BNP) levels negatively impacts activity of daily living in patients with hip fracture

**DOI:** 10.1371/journal.pone.0237387

**Published:** 2020-08-13

**Authors:** Yusuke Tamamura, Micihko Matsuura, Sumiko Shiba, Toshio Nishikimi

**Affiliations:** 1 Department of Rehabilitation, Wakakusa Tatsuma Rehabilitation Hospital, Daito City, Osaka, Japan; 2 Department of Physical Therapy, Konan Women’s University, Higashinada-ku, Kobe City, Hyogo; 3 Department of Medicine, Wakakusa Tatsuma Rehabilitation Hospital, Daito City, Osaka, Japan; 4 Department of Cardiovascular Medicine, Kyoto University Graduate School of Medicine, Sakyo-ku, Kyoto, Japan; International University of Health and Welfare, School of Medicine, JAPAN

## Abstract

Several studies have shown that nutrition and muscle strength were associated with functional recovery in patients with hip fracture. However, the impact of heart failure on the improvement of activity of daily living (ADL) in patients with hip fracture have not been fully investigated. The purpose was investigating the effect of heart failure on the ADL improvement by rehabilitation in patients with hip fracture. A total of 116 patients with hip fracture discharged from our convalescent rehabilitation ward were studied. Heart failure was assessed based on plasma B-type natriuretic peptide (BNP) levels on admission. ADL was assessed based on rehabilitation effectiveness (REs), which was calculated using the FIM instrument. Clinical, demographic, and nutritional variables were measured. Multiple regression analysis was performed with REs as the dependent variable; variables showing significant correlation with REs in univariate analyses were selected as independent variables. Based on plasma BNP levels, we assigned 39 patients to a Low group: 22 (17−25) median (interquartile) pg/mL, 39 to a Middle group: 52 (42−65) pg/mL, and 38 to a High group: 138 (93−209) pg/mL. REs, handgrip strength, Hb, albumin, and GNRI were higher and age was younger in the Low group than High group (each p < 0.01, respectively). Multiple linear regression analysis revealed that age (p < 0.05), sex (p < 0.05), handgrip strength (p < 0.01), FOIS at admission (p < 0.01), rehabilitation time per day (p < 0.01), and BNP (p < 0.05) were significantly associated with REs. The effect of rehabilitation on ADL improvement was significantly blunted in the High group compared to the Low group. In conclusion, these results suggest that heart failure assessed based on plasma BNP levels negatively impacts improvements in ADL achieved through rehabilitation in patients with hip fracture.

## Introduction

Hip fractures in older adults have critical consequences in terms of increased risk of mortality, functional decline, and decreased quality of life. [1–3] Globally, the annual number of hip fractures was estimated to be 1.6 million in 2000, and this number is expected to rise to around 6.3 million by 2050. [4] This is particularly noteworthy, as an earlier study estimated that 60% of patients with hip fracture do not recover previous functional ability. [5] Moreover, about 40% of these patients are unable to return to their homes after acute rehabilitation. [6] This suggests that many hip fracture patients suffer a significant loss of functional independence, and that functional recovery is one of the most important rehabilitation goals for patients with hip fractures. For that reason, identification of predictors of functional status is important. For example, several studies have shown that handgrip strength, nutritional status, and the presence of diabetes mellitus are prognostic factors for hip fracture patients. [7–9]

In developed countries, the populations are rapidly aging, and the numbers of heart failure patients are increasing as these populations age. [10] In addition, a recent large-scale observational study reported that about 30% of heart failure patients are over 80 years old. [11] This suggests that the number of hip fractures accompanied by heart failure will greatly increase in the future. However, the impact of heart failure on the recovery of activity of daily living (ADL) in patients following hip fracture has not been fully investigated. A previous study showed that improvement of the functional independence measurement (FIM) during the first 6 months was diminished in elderly hip fracture patients with a self-reported history of heart failure. [12] Similarly, FIM effectiveness was reportedly reduced in hip fracture patients with a history of heart failure. [13] However, because these two studies relied on self-reported diagnoses of heart failure or retrospectively confirmed heart failure diagnosis based on the medical record, the degree and severity of heart failure during rehabilitation could not be assessed.

B-type natriuretic peptide (BNP) is a cardiac hormone produced and secreted by the heart. [14] Plasma BNP levels increase in proportion to the severity of heart failure and they decrease as treatment improves the patient’s condition. [15] Since the signs and symptoms of heart failure are often nonspecific, a helpful history is not often obtainable, and dyspnea may be a nonspecific finding in whom comorbidity with respiratory disease is common, it is often difficult to diagnose heart failure especially in elderly patients. In this clinical setting, BNP is often superior to clinical judgement for diagnosing heart failure. [16,17] In the present study, we examined the effect of heart failure on ADL improvement through rehabilitation in hip fracture patients. Measurements of BNP enabled us to assess the current state of the patients’ heart failure without relying on the history of heart failure.

## Methods

### Ethics

This study was approved by the ethics committee of Wakakusa-Tatsuma Rehabilitation Hospital (approved number 19100761). All participants or their legal representatives provided written informed consent. The study was performed in accordance with the ethical standards of the 1964 Declaration of Helsinki and its later amendments.

### Study design and subject

**Fig 1:**
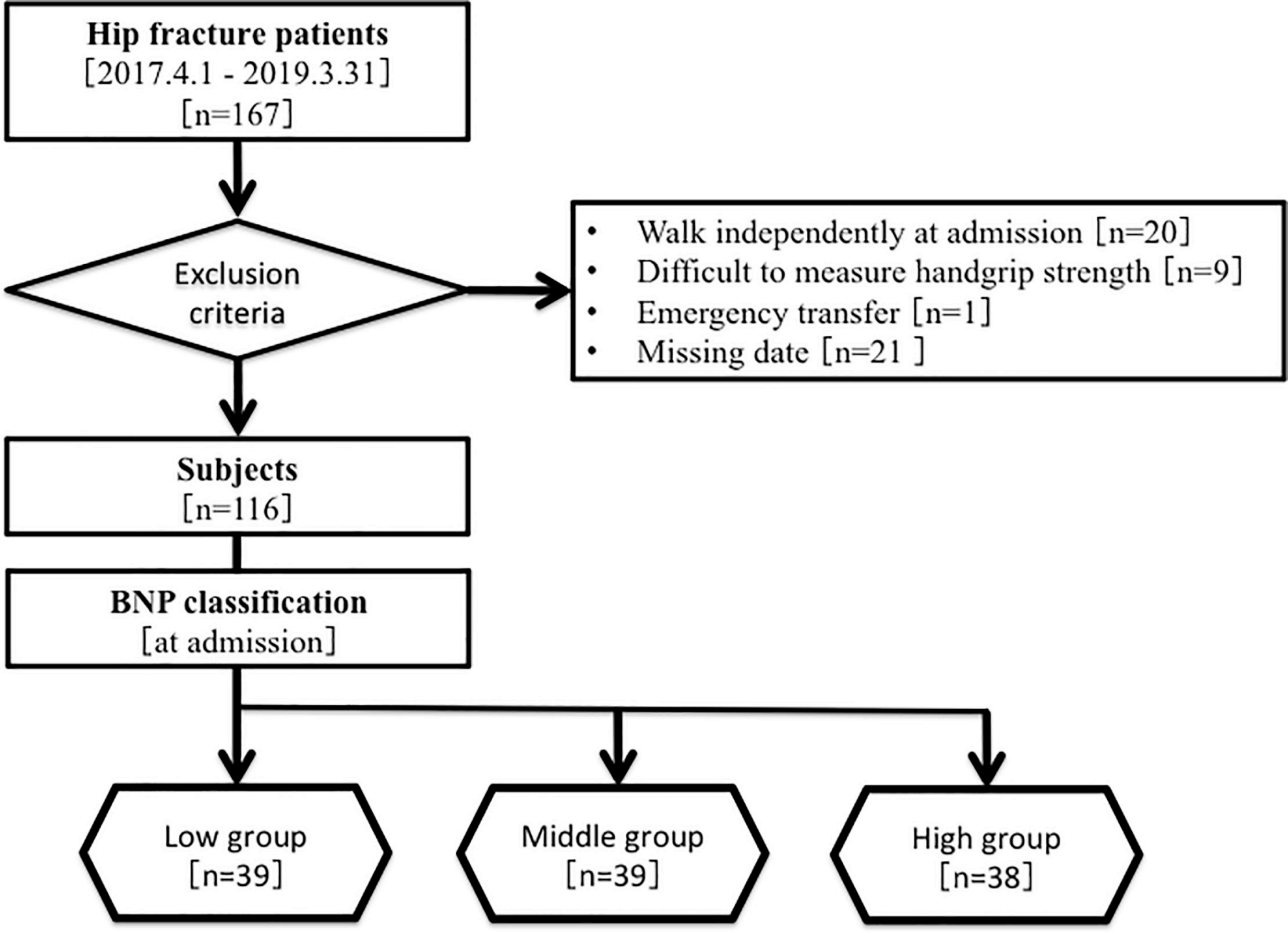
Flowchart of subject selection and classification. BNP = B-type natriuretic peptide.

This study was a retrospective observational cohort study. A total of 167 patients (age ≥ 65 years) were discharged from the convalescent rehabilitation ward of Wakakusa-Tatusma Rehabilitation Hospital after surgery for hip fracture between April 1, 2017 and March 31, 2019. The hip fractures included femoral neck, trochanteric, and subtrochanteric fractures. After exclusion of 51 patients, 116 patients were studied (Fig 1). The subjects underwent physical or occupational therapy from 40 to 180 minutes per day in a convalescent rehabilitation ward. In Japan, the medical insurance system allows hip fracture patients to be transferred from an acute care hospital to a convalescent rehabilitation ward within approximately 30 days after injury and to receive rehabilitation for up to 90 days after admission to the convalescent rehabilitation hospital. [18]

### Measurements

Demographic data including age, gender, height, weight, body mass index, period from injury to hospitalization, and length of hospital stay (LOS) were collected from medical records. Clinical data such as swallowing function, nutritional status, and rehabilitation index were also collected. The swallowing function was evaluated using the functional oral intake scale (FOIS). Nutritional status was assessed based on serum albumin levels and the geriatric nutritional risk index (GNRI). The rehabilitation index was the time of rehabilitation per day.

We used the FIM as the ADL indicator. This tool is among the most widely used measurements for assessing a patient’s ability to perform ADL and consists of 13 motor and 5 cognitive items that are each scored 1–7, depending on the amount of assistance required to perform each basic activity. The motor items include eating, grooming, bathing, dressing the upper body and lower body, toileting, bladder and bowel management, bed, chair, or wheelchair transfer, toilet transfer, tub transfer, walk/wheelchair, and use of the stairs. The five cognitive items include comprehension, expression, social interaction, problem solving, and memory. The total FIM score ranged from 18 (reflecting full dependence) to 126 (reflecting complete independence). [19]

On admission, physical examination, electrocardiogram, chest X-ray, and blood sampling for BNP and C-reactive protein (CRP) were performed. Cardiothoracic ratio was measured by chest X-ray. CRP was measured as an indicator of inflammation, while BNP was measured as an indicator of heart failure status. From the medical history including history taking from patients and their family, patient's symptom, medical letter of referral, and above findings on admission, the patients were classified to the heart failure stage according to the ACC/AHA guideline [20] by an experienced cardiologist who specializes heart failure (T.N.). Plasma BNP levels were measured using a sandwich chemiluminescence enzyme immunoassay [21]. We divided the patients into three groups based on their plasma BNP levels at admission: 39 were in the Low group, 39 in the Middle group, and 38 in the High group, starting from the lowest BNP at admission.

### Main outcome

The main outcome was rehabilitation effectiveness (REs) calculated using the FIM instrument, which was calculated using the following formula: (FIM at discharge − FIM at admission) / (126 –FIM at admission). REs was expressed as a percentage reflecting the proportion of potential improvement actually achieved during rehabilitation, thus REs correcting for a ceiling effect. REs is the concept first suggested by Heinemann et al. [22] as achievement of rehabilitation potential. According to a recent review paper, the concept of 'achievement of rehabilitation potential' is the same as the Montebello Rehabilitation Factor Score (MRFS), relative functional gain (RFG), and REs, and we used REs in this study because REs is currently the most widely used [23].

### Statistical analysis

Continuous data are presented as means ± standard deviation, while non-parametric data are presented as the median (interquartile range 25–75 percentile). Differences among groups were evaluated using one-way analysis of variance (ANOVA) with post-hoc Fisher’s PLSD test. Categorical data are expressed as incidences and percentages, and comparisons were made using the χ^2^-test. Multiple regression analysis was performed with REs as the dependent variable; age, gender, handgrip strength as an indicator of physical function, FOIS at admission as an indicator of swallowing status, GNRI as an indicator of nutrition status, provided rehabilitation time as an indicator of rehabilitation, and variables that had a significant correlation in the univariate regression analysis of REs were selected as independent variables. FIM scores and serum albumin levels were excluded because they closely correlated with REs or GNRI, respectively. In addition, analysis of covariance (ANCOVA) model was estimated to examine the difference in REs by BNP levels, controlling foe age and sex. P values < 0.05 were considered statistically significant. Statistical analyses were performed using STATVIEW version 5 (Abacus Concepts, Berkeley, CA).

## Results

Among the 116 hip fracture patients studied, the mean age was 82 ± 8 years (range: 65 to 102 years), and 26 patients (22%) were men. Based on their plasma BNP levels at admission, we assigned 39 patients to the Low group (median: 22 pg/mL, interquartile: 17−25 pg/mL), 39 patients to the Middle group (median: 52 pg/mL, interquartile: 42−65 pg/mL), and 38 patients to the High group (median: 138 pg/mL, interquartile: 93−209 pg/mL). According to the ACC/AHA guideline classification, HF Stage A was 20.7% (24/116) and percentage of heart failure risk factors was hypertension (recorded in medical reports, antihypertensive treatment on admission, or systolic blood pressure >140mmHg or diastolic blood pressure >90 mmHg on admission) was 66.7% (16/24), diabetes mellitus (recorded in medical reports, recent use of antidiabetic drugs, a fasting blood glucose value of ≥126 mg/dL on admission, and/or a hemoglobin A1c value of ≥ 6.5% on admission) was 20.8% (5/24), and others were 12.5% (3/24). HF Stage B was 48.3% (56/116) and percentage of patients with structural heart disease was left ventricular hypertrophy (by echocardiography or electrocardiogram) was 69.6% (39/56), cardiac dilatation (by echocardiography or thoracic computed tomography or chest X-ray) was 16.1% (9/56), decrease of left ventricular ejection fraction (ejection fraction < 50% by echocardiography) was 10.7% (6/56), and others were 3.6% (2/56). HF Stage C was 28.4% (33/116) and a history of heart failure was obtained from medical referral letter (13/33), from the patients or their family (11/33), from the review of patients medical record (7/33), and documentation of signs and symptom of heart failure by patient’s physician on admission (2/33). Most of the patients were in NYHA functional class II (NYHA I/II/III: 5/22/6). Stage 0 was 2.6% (3/116). The classification of each group is as follows: LOW group: 21 patients were HF Stage A, 15 patients were HF Stage B, 1 patient was HF Stage C, and 2 were Stage 0. Middle group: 2 patients were HF Stage A, 29 patients were HF Stage B, 7 patients were HF Stage C, and 1 was Stage 0. High group: 1 patient was HF Stage A, 12 patients were HF Stage B, and 25 patients were HF Stage C. The demographic and clinical data for each group are presented in Table 1.

**Table 1:** Demographic and clinical data for each BNP group.

Variable	Total (n = 116)	Low (n = 39)	Middle (n = 39)	High (n = 38)	P value
BNP (pg/ml) median (25–75%)	50 (25–92)	22 (17–25)	52 (42–65)	138 (93–209)	
BNP (pg/ml) mean ± SD	91 ± 139	21 ± 7	52 ± 13	204 ± 200*†	< 0.01
HF Stage, n (%)					
0	3 (2.6)	2 (5.1)	1 (2.6)	0 (0)	
A	24 (20.7)	21 (53.8)	2 (5.1)	1 (2.6)	< 0.01
B	56 (48.3)	15 (38.5)	29 (74.4)	12 (31.6)	< 0.01
C	33 (28.4)	1 (2.6)	7 (17.9)	25 (65.8)	< 0.01
D	0 (0)	0 (0)	0 (0)	0 (0)	
CTR (%)	55.8 ± 6.2	51.7 ± 4.8	55.7 ± 5.2*	60.0 ± 5.5*†	< 0.01
Age (years)	82 ± 8	79 ± 8	81 ± 7	84 ± 10 *	< 0.05
Male, n, (%)	26 (22)	12 (31)	8 (21)	6 (16)	0.27
Period from injury to hospitalization (day)	26 ± 11	25 ± 10	27 ± 12	27 ± 12	0.71
Length of hospital stay (days)	75 ± 19	75 ± 15	75 ± 18	74 ± 22	0.89
Height (cm)	150.5 ± 9.6	152.3 ± 10.1	150.6 ± 8.4	148.7 ± 9.9	0.25
Weight at admission (kg)	46.4 ± 9.6	48.4 ± 10.7	46.5 ± 7.6	44.2 ± 9.9	0.17
BMI at admission (kg/m^2^)	20.4 ± 3.6	20.8 ± 4.1	20.7 ± 3.5	19.9 ± 3.2	0.49
Handgrip strength (kg)	11.9 ± 7.5	14.8 ± 8.4	9.9 ± 4.1*	10.8 ± 8.3*	< 0.01
FOIS at admission	6.0 ± 1.1	6.2 ± 1.0	6.0 ± 1.0	5.7 ± 1.2	0.25
FIM at admission (score)					
Motor FIM	35 ± 13	38 ± 13	34 ± 14	34 ± 13	0.39
Cognitive FIM	20 ± 7	22 ± 7	21 ± 7	18 ± 7*	< 0.05
Total FIM	56 ± 18	60 ± 18	55 ± 19	52 ± 18	0.17
Provided energy (kcal/day)	1578 ± 213	1604 ± 180	1566 ± 266	1562 ± 184	0.64
Albumin at admission (mg/dl)	3.3 ± 0.6	3.5 ± 0.5	3.2 ± 0.6*	3.2 ± 0.6*	< 0.05
GNRI at admission	88.2 ± 11.8	92.1 ± 12.1	86.9 ± 12.4*	85.6 ± 10.0*	< 0.05
CHE at admission (IU/l)	204 ± 75	233 ± 53	200 ± 56*	177 ± 49*	< 0.01
CRP at admission (mg/dl)	0.9 ± 1.5	0.8 ± 1.7	1.1 ± 1.9	0.7 ± 0.8	0.43
Hb at admission (g/dl)	10.9 ± 1.4	11.5 ± 1.6	10.7 ± 1.4*	10.4 ± 1.1*	< 0.01
Cre at admission (mg/dl)	0.8 ± 0.4	0.7 ± 0.3	0.8 ± 0.5	0.8 ± 0.3	0.71
eGFR at admission (mL/min/1.73 m^2^)	68.1 ± 24.7	71.4 ± 23.4	66.5 ± 25.9	60.4 ± 25.2	0.61
Weight at discharge (kg)	46.9 ± 9.4	49.2 ± 9.9	46.4 ± 7.1	45.0 ± 10.7*	0.13
BMI at discharge (kg/m^2^)	20.6 ± 3.5	21.2 ± 3.9	20.5 ± 3.3	20.2 ± 3.3	0.42
FOIS at discharge	5.9 ± 1.2	6.1 ± 1.0	5.9 ± 1.3	5.7 ± 1.2	0.25
FIM at discharge (score)					
Motor FIM	61 ± 19	67 ± 19	61 ± 18	54 ± 20*	< 0.05
Cognitive FIM	23 ± 7	26 ± 7	23 ± 7	21 ± 8*	< 0.05
Total FIM	84 ± 26	93 ± 24	84 ± 24	76 ± 27*	< 0.05
Albumin at discharge (mg/dl)	3.4 ± 0.5	3.6 ± 0.5	3.3 ± 0.5*	3.3 ± 0.4*	< 0.05
GNRI at discharge	89.7 ± 11.4	93.2 ± 12.4	88.3 ± 10.1	87.5 ± 11.0*	0.06
CHE at discharge (IU/l)	213 ± 57	240 ± 48	202 ± 60*	197 ± 53*	< 0.01
CRP at discharge (mg/dl)	0.9 ± 2.1	0.4 ± 0.6	1.3 ± 2.0	1.1 ± 2.9	0.13
Rehabilitation outcome					
Rehabilitation time/day (min)	120 ± 16	124 ± 14	116 ± 16*	120 ± 14	< 0.05
Motor FIM gain (score)	25 ± 14	29 ± 13	27 ± 13	20 ± 15*†	< 0.05
Total FIM gain (score)	28 ± 16	33 ± 15	29 ± 14	23 ± 18*	< 0.05
Motor FIM effectiveness (%)	48.6 ± 28.3	58.5 ± 26.9	49.6 ± 23.8	37.8 ± 30.8*	< 0.01
REs (%)	44.2 ± 26.4	53.5 ± 25.5	44.4 ± 22.8	34.8 ± 28.2*	< 0.01
Discharge to home (%)	62 (73/116)	74 (29/39)	67 (26/39)	47 (18/38)	< 0.05

Data are presented as the mean ± standard deviation. Statistical analysis: one-way ANOVA with post-hoc Fisher’s PLSD test.χ2-test. Significant Difference: p<0.05 *) vs. the Low Group, †) vs. the Middle Group.

BMI = body mass index; BNP = B-type natriuretic peptide; CHE = cholinesterase; Cre = creatinine; CRP = plasma C-reactive protein; CTR = cardio thoracic ratio; eGFR = estimated glomerular filtration rate; FIM = functional independence measure; FOIS = functional oral intake scale; GNRI = geriatric nutritional risk index, Hb = hemoglobin; REs; rehabilitation effectiveness.

**Fig 2:**
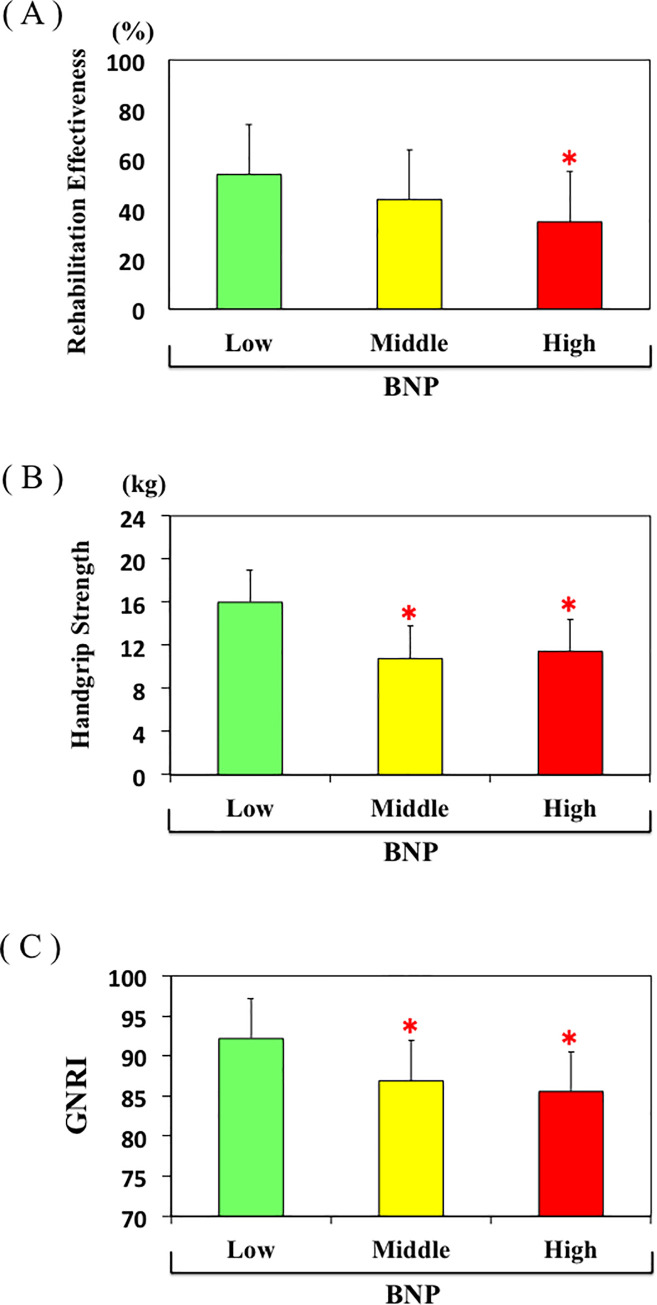
A, B, C. Rehabilitation effectiveness (A), handgrip strength (B), and GNRI (C) in the Low, Middle, and High plasma BNP groups. *p<0.05 vs. Low plasma BNP group.

**Fig 3:**
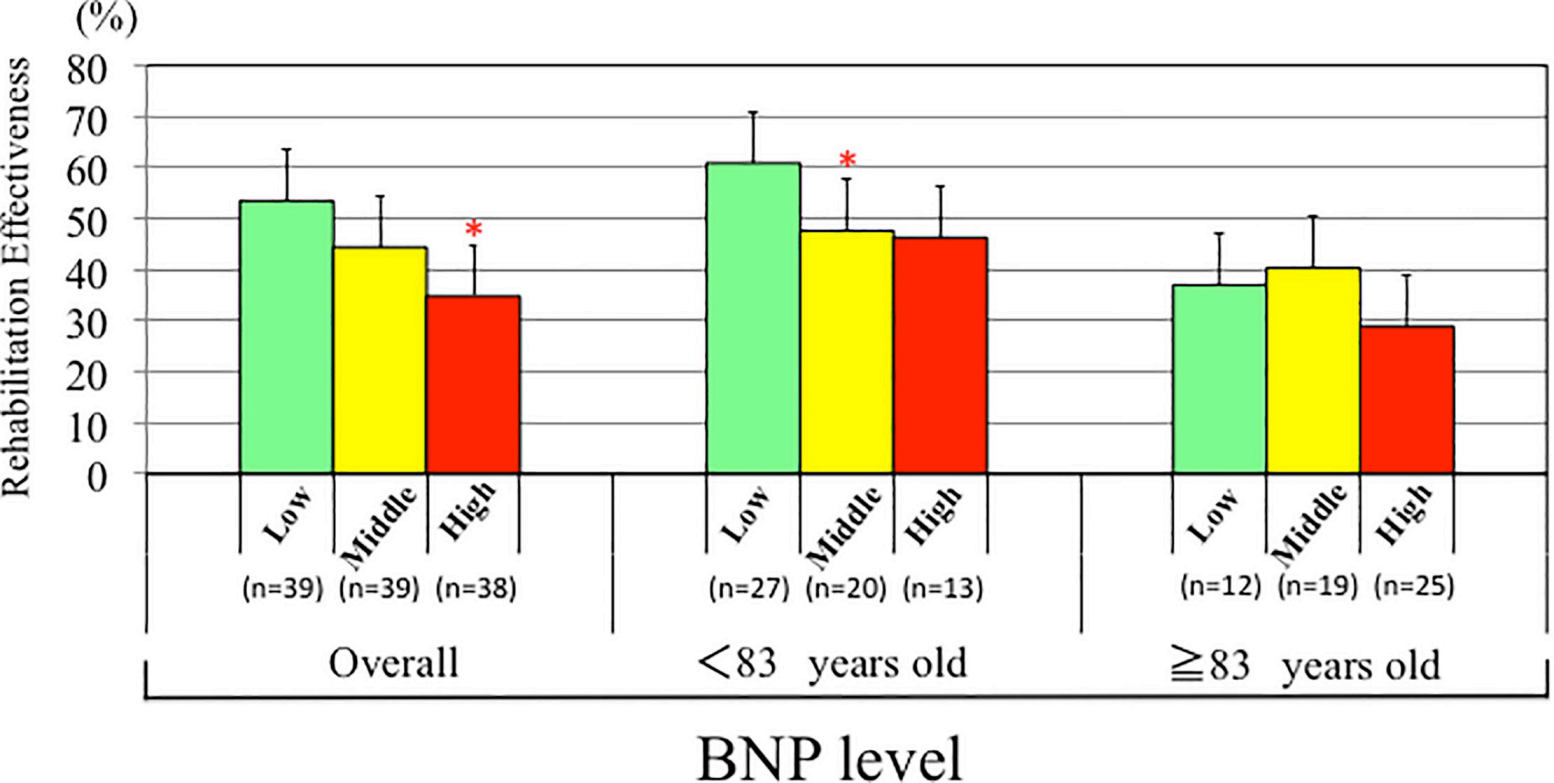
Distribution of REs values according to age-specific tertiles of plasma BNP levels overall, in patients <83 years old and patients ≥ 83 years old, respectively. *p<0.05 vs. Low plasma BNP group.

The mean age in the Low group was younger than in the High group, whereas there were no differences with respect to gender, period from injury to hospitalization, or LOS among the groups. Handgrip strength, cognitive FIM, albumin, GNRI, cholinesterase, and hemoglobin at admission were all higher in the Low group than the High group (Fig 2). In addition, motor FIM, cognitive FIM, total FIM, albumin, GNRI, and cholinesterase at discharge were also higher in Low group than the High group. Regarding outcome, motor FIM gain, total FIM gain, motor FIM effectiveness, percentage discharged to home, and REs were all higher in the Low group than the High group (Fig 2). After adjusted age and sex by ANCOVA, mean values of REs decreased significantly from the Low to the High tertile of BNP (P<0.01). According to age-specific tertiles of BNP, a significant difference was observed between Low and Middle tertile of BNP in patients < 83 years old group, but not in patients ≥ 83 years old group (Fig 3). There was no difference in rehabilitation time/day between the two groups. Most of the values in the Middle group were between those in the Low and High groups, though some of the values in the Middle group significantly differed from those in Low group.

We also analyzed the data after dividing the patients into three groups based on the REs tertiles. Demographic and clinical data for each group is presented in Table 2.

**Table 2:** Demographic and clinical data for each REs group.

Variable	Total (n = 116)	Low (n = 39)	Middle (n = 39)	High (n = 38)	P value
REs (%)	44.2 ± 26.4	15.2 ± 17.0	45.5 ± 6.1*	72.7 ± 10.7*†	< 0.01
BNP (pg/ml) mean ± SD	91 ± 139	151 ± 217	69 ± 61*	54 ± 48*	< 0.01
HF Stage, n (%)					
0	3 (2.6)	1 (2.6)	2 (5.2)	0 (0)	
A	24 (20.7)	9 (23.0)	6 (15.3)	9 (23.7)	0.86
B	56 (48.3)	10 (25.6)	23 (59.0)	23 (60.5)	< 0.01
C	33 (28.4)	19 (48.8)	8 (20.5)	6 (15.8)	< 0.01
D	0 (0)	0 (0)	0 (0)	0 (0)	
CTR (%)	55.8 ± 6.2	57.1 ± 7.1	55.3 ± 5.4	54.8 ± 6.0	0.24
Age (years)	82 ± 8	85 ± 8	81 ± 8*	78 ± 7 *	< 0.01
Male, n, (%)	26 (22)	8 (21)	9 (23)	9 (24)	0.94
Period from injury to hospitalization (day)	26 ± 11	28 ± 12	25 ± 11	27 ± 10	0.40
Length of hospital stay (days)	75 ± 19	72 ± 21	79 ± 18	73 ± 16	0.16
Height (cm)	150.5 ± 9.6	146.4 ± 9.4	153.2 ± 9.1*	152.2 ± 9.1*	< 0.01
Weight at admission (kg)	46.4 ± 9.6	42.9 ± 9.0	47.1 ± 9.4	49.4 ± 9.3*	< 0.01
BMI at admission (kg/m^2^)	20.4 ± 3.6	20.0 ± 3.4	20.1 ± 3.8	21.3 ± 3.7	0.23
Handgrip strength (kg)	11.9 ± 7.5	8.7 ± 5.2	13.2 ± 6.9*	16.6 ± 7.7*†	< 0.01
FOIS at admission	6.0 ± 1.1	5.4 ± 1.1	6.1 ± 1.1*	6.5 ± 0.6*	< 0.01
FIM at admission (score)					
Motor FIM	35 ± 13	29 ± 10	35 ± 14*	43 ± 12*†	< 0.01
Cognitive FIM	20 ± 7	15 ± 7	21 ± 5*	25 ± 5*†	< 0.01
Total FIM	56 ± 18	43 ± 15	56 ± 17*	68 ± 15*†	< 0.01
Provided energy (kcal/day)	1578 ± 213	1584 ± 219	1590 ± 201	1559 ± 222	0.80
Albumin at admission (mg/dl)	3.3 ± 0.6	3.2 ± 0.6	3.3 ± 0.5	3.5 ± 0.7*	< 0.05
GNRI at admission	88.2 ± 11.8	84.7 ± 10.8	87.8 ± 9.9	92.2 ± 13.5*	< 0.05
CHE at admission (IU/l)	204 ± 75	180 ± 57	204 ± 48	227 ± 58*	< 0.05
CRP at admission (mg/dl)	0.9 ± 1.5	1.2 ± 1.9	1.0 ± 1.8	0.4 ± 0.7	0.11
Hb at admission (g/dl)	10.9 ± 1.4	10.5 ± 1.4	10.7 ± 1.2	11.5 ± 1.7*†	< 0.01
Cre at admission (mg/dl)	0.8 ± 0.4	0.7 ± 0.3	0.8 ± 0.4	0.8 ± 0.5	0.31
eGFR at admission (mL/min/1.73 m^2^)	68.1 ± 24.7	71.6 ± 23.9	68.6 ± 25.0	64.1 ± 25.4	0.41
Weight at discharge (kg)	46.9 ± 9.4	42.9 ± 8.0	48.1 ± 9.7*	49.7 ± 9.3*	< 0.01
BMI at discharge (kg/m^2^)	20.6 ± 3.5	20.0 ± 3.2	20.5 ± 3.7	21.4 ± 3.6	0.23
FOIS at discharge	5.9 ± 1.2	5.2 ± 1.4	6.1 ± 1.0*	6.4 ± 0.7*	< 0.01
FIM at discharge (score)					
Motor FIM	61 ± 19	39 ± 14	64 ± 8*	79 ± 8*†	< 0.01
Cognitive FIM	23 ± 7	17 ± 6	24 ± 5*	30 ± 4*†	< 0.01
Total FIM	84 ± 26	56 ± 18	87 ± 11*	109 ± 9*†	< 0.01
Albumin at discharge (mg/dl)	3.4 ± 0.5	3.2 ± 0.5	3.5 ± 0.5*	3.5 ± 0.5*	< 0.01
GNRI at discharge	89.7 ± 11.4	85.3 ± 11.6	90.6 ± 10.9*	93.3 ± 10.5*	< 0.01
CHE at discharge (IU/l)	213 ± 57	193 ± 64	215 ± 51	231 ± 49*	< 0.05
CRP at discharge (mg/dl)	0.9 ± 2.1	1.5 ± 3.0	0.8 ± 1.2	0.5 ± 1.4*	0.12
Rehabilitation outcome					
Rehabilitation time/day (min)	120 ± 16	116 ± 18	122 ± 14	123 ± 13*	0.08
Motor FIM gain (score)	25 ± 14	11 ± 11	29 ± 8*	36 ± 8*†	< 0.01
Total FIM gain (score)	28 ± 16	12 ± 13	32 ± 8*	41 ± 9*†	< 0.01
Motor FIM effectiveness (%)	48.6 ± 28.3	17.7 ± 19.6	51.6 ± 8.9*	77.3 ± 12.3*†	< 0.01
Discharge to home (%)	62 (73/116)	31 (12/39)	67 (26/39)	89 (18/38)	< 0.01

Data are presented as the mean ± standard deviation. Statistical analysis: one-way ANOVA with post-hoc Fisher’s PLSD test. χ2-test. Significant Difference: p<0.05 *) vs. the Low Group, †) vs. the Middle Group.

BMI = body mass index; BNP = B-type natriuretic peptide; CHE = cholinesterase; Cre = creatinine; CRP = plasma C-reactive protein; CTR = cardio thoracic ratio; eGFR = estimated glomerular filtration rate; FIM = functional independence measure; FOIS = functional oral intake scale; GNRI = geriatric nutritional risk index, Hb = hemoglobin; REs, rehabilitation effectiveness.

**Fig 4:**
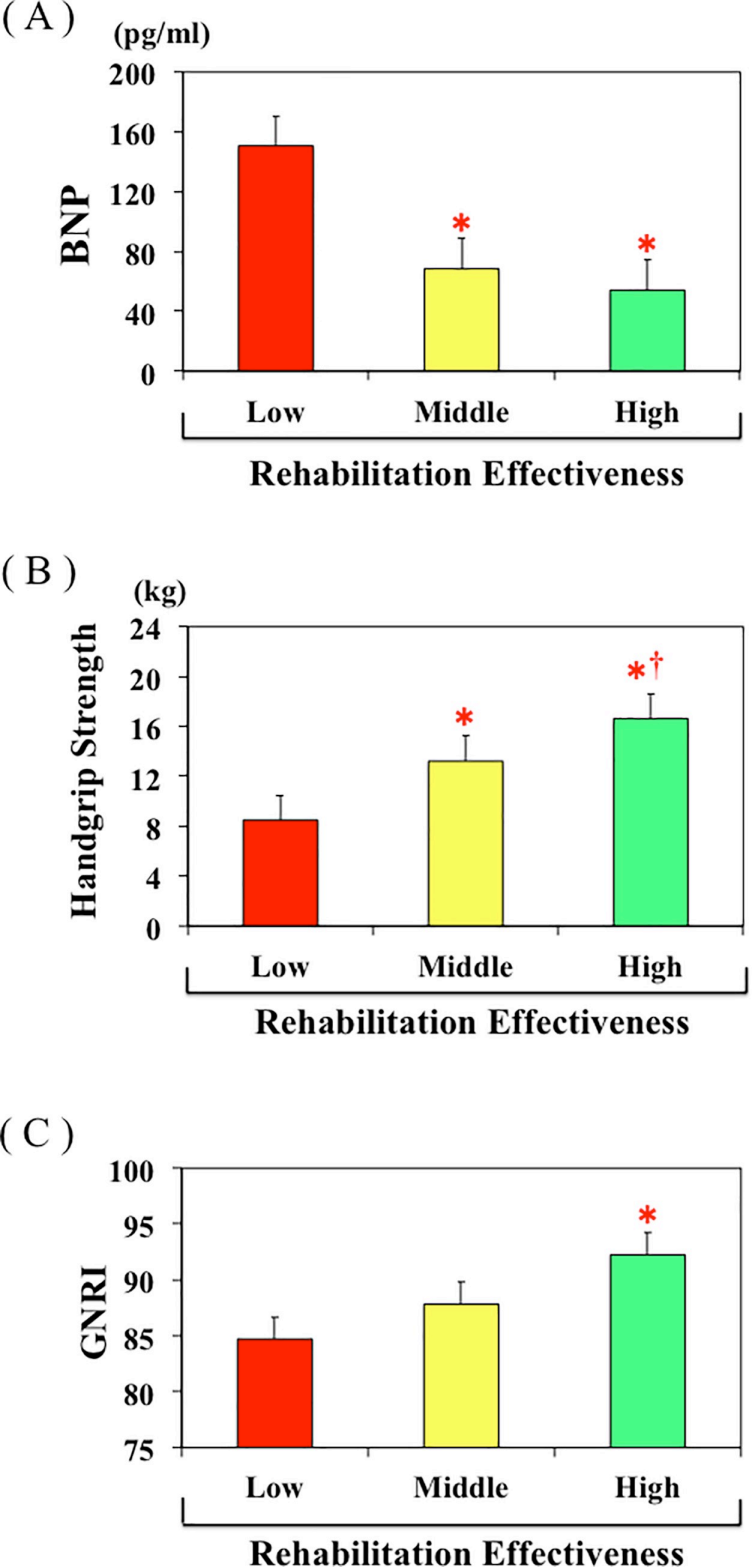
A, B, C. Plasma BNP levels (A), handgrip strength (B), and GNRI (C) in the Low, Middle, and High Res groups. *p<0.05 vs. Low REs group, †p<0.05 vs. Middle REs group.

The mean age in the High group was younger than in the Low group, whereas there were no differences with respect to gender, period from injury to hospitalization, or LOS among the three groups. Height, weight, handgrip strength, FOIS, motor FIM, cognitive FIM, total FIM, albumin, GNRI, cholinesterase, and hemoglobin at admission were all significantly higher in the High group than the Low group, while plasma BNP at admission was lower in the High group than the Low group (Fig 4). In addition, weight, FOIS, motor FIM, cognitive FIM, total FIM, albumin, GNRI, and cholinesterase at discharge were all higher in the High group than the Low group, whereas plasma CRP level at discharge was lower in the High group than the Low group. Regarding heart failure stage, the proportion of HF stage C was significantly higher in Low group. Regarding outcomes, motor FIM gain, total FIM gain, motor FIM effectiveness, and percentage discharged to home were all higher in the High group than the Low group. There were no differences in rehabilitation time/day between the two groups. Most of the values in the Middle group fell between those in the Low and High groups, though some values in the Middle group significantly differed from those in the Low group.

Univariate analyses showed that age (r = -0.408, p<0.01), handgrip strength at admission (r = 0.455, p<0.01), FOIS at admission (r = 0.416, p<0.01), GNRI at admission (r = 0.334, p<0.01), albumin at admission (r = 0.322, p<0.01), BNP at admission (r = -0.303, p<0.01), and provided rehabilitation time per day (r = 0.291, p<0.01) were all significantly associated with REs. Multiple linear regression analysis revealed that age (p<0.05), sex (p<0.05), handgrip strength (p<0.01), FOIS at admission (p<0.01), provided rehabilitation time per day (p<0.01), and BNP (p<0.05) were all significantly associated with REs (Table 3).

**Table 3:** Multiple linear regression analysis for REs in 116 patients with hip fracture in convalescent rehabilitation wards in Japan using demographic, clinical, biochemical date.

		95% CI		
	β	Lower	Higher	P value
(Constant)	-0.017	-0.176	0.682	0.96
age	-0.19	-0.011	-0.001	<0.05
sex	-0.187	-0.23	-0.006	<0.05
Handgrip Strength	0.36	0.006	0.02	<0.01
FOIS at admission	0.244	0.02	0.098	<0.01
GNRI at admission	-0.022	-0.004	0.003	0.80
Hb at admission	0.056	-0.021	0.042	0.51
BNP at admission	-0.151	-0.001	< -0.001	<0.05
Rehabilitation time/day	0.208	0.021	0.123	<0.01

BNP = B-type natriuretic peptide; FOIS = functional oral intake scale; GNRI = geriatric nutritional risk index; Hb = hemoglobin.

**Fig 5:**
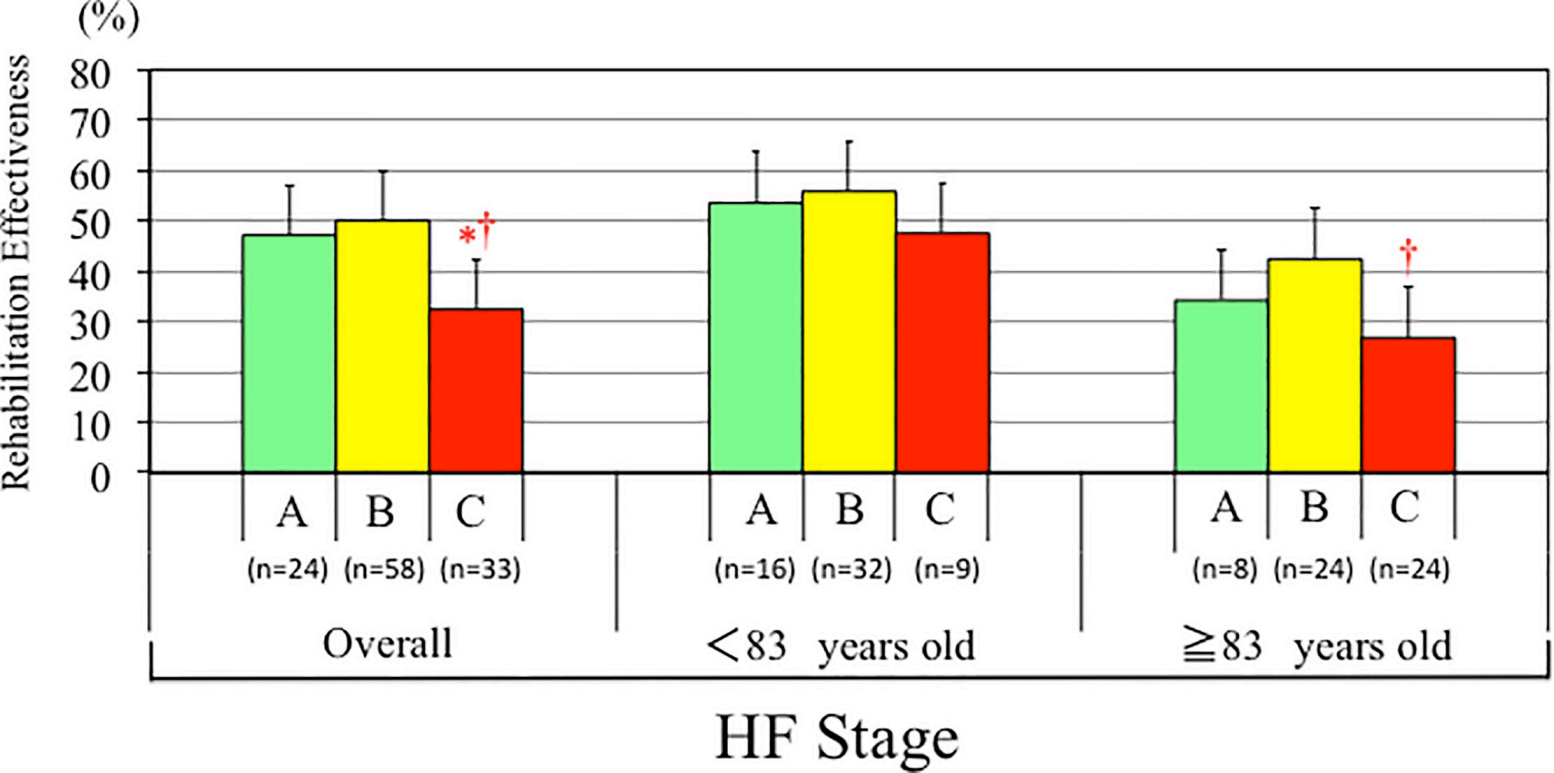
Distribution of REs values according to age-specific heart failure stage according to the ACC/AHA guideline classification overall, in patients <83 years old and patients ≥ 83 years old, respectively. *p<0.05 vs. HF Stage A group. †p<0.05 vs. HF Stage B group.

We also analyzed the effect of heart failure stage according to the ACC/AHA guideline classification on REs. After controlling age and sex by ANCOVA, mean values of REs decreased significantly from the HF stage A to HF stage C (P<0.05). According to age-specific HF stage classification, a significant difference was observed between HF stage A and HF stage B in patients < 83 years old group, but no difference among the groups in patients ≤ 83 years old (Fig 5).

## Discussion

In the present study, we showed for the first time that heart failure status assessed using plasma BNP levels as an indicator negatively impacts ADL improvement through rehabilitation after hip fracture.

BNP is a hormone secreted by the heart, mainly from the ventricles. Plasma BNP levels increase in proportion to the severity of heart failure [14] and decrease when treatment improves a heart failure patient’s condition. [15] Guidelines from the European Society of Cardiology and American College of Cardiology/American Heart Association recommend BNP measurement when diagnosing heart failure, as it well reflects the status of the disease at the time of the measurement [24,25]. Indeed, in this clinical setting, BNP is often superior to clinical judgement for diagnosing heart failure. [16,17] In the present study, we divided the participants into groups based on their plasma BNP concentration tertiles. The first tertile had median plasma BNP concentration of 22 pg/mL and an interquartile of 17–25 pg/mL, which is close to normal according to the BNP guidelines from the Japanese Society for Heart Failure. [26] Those guidelines also state that the plasma BNP concentrations in the second tertile (median: 52 pg/mL, interquartile: 42–65 pg/mL) indicate asymptomatic heart disease such as left ventricular hypertrophy, asymptomatic valvular heart disease, or ischemic heart disease, while the plasma BNP concentrations in the third tertile (median: 138 pg/mL, interquartile: 93–209 pg/mL) indicate latent heart failure. [26] In this study, we classified the patients into heart failure stage classification according to ACC/AHA guideline, and we compared it with BNP tertile. The BNP Low group mainly consisted of stage A patients, the BNP Middle group mainly consisted of stage B patients, and the BNP High group mainly consisted of stage C patients. These results indicate that classification by BNP tertile well reflects the classification of heart failure stage by ACC/AHA guideline. These results suggest that about half of hip fracture patients have structural heart disease without symptom of heart failure and a quarter patients have symptomatic heart failure with structural heart disease. Thus, it is very important to catch the heart failure status based on plasma BNP levels on admission in patients with hip fracture to plan rehabilitation program.

An earlier study showed that dyspnea on mild exertion due to heart failure is a negative independent factor associated with rehabilitation outcome in patients with hip fracture. [27] Those authors suggest that the severe functional impairment caused by heart failure, but not the disease per se, is associated with rehabilitation outcome after hip fracture. Mathew et al. [12] also reported that maximum functional improvement occurs during the first 6 months after rehabilitation in a self-reporting heart failure group, and that this group had a lower level of recovery of transfer ability and locomotion than a group without disease. Itagaki et al. [13] recently reported that hip fracture patients with heart failure showed lower REs during hospitalization than those without heart failure, even after adjustment for confounding factors. These three studies appear to indicate that heart failure negatively affects the ADL improvement achieved through rehabilitation; however, the diagnosis of heart failure in the first two studies was based on self-reported histories, and the diagnosis of heart failure in the third study was retrospectively confirmed based on medical records. Because heart failure conditions change daily [28] and often greatly improve with pharmacotherapy [29], the degree to which heart failure status actually affected changes in ADL is unclear in these studies.

In the present study, we divided patients into groups based on their plasma BNP levels on admission, which reflected their heart failure status at that time. Notably, the hip fracture patients in the highest plasma BNP tertile were older and scored lower in the handgrip strength test, cognitive FIM, hemoglobin, serum albumin, and GNRI than patients in the first tertile. Taken together, those results indicate that the hip fracture patients in the third plasma BNP tertile were older, had poorer cognition, poorer nutrition, and were more anemic than the patients in the first tertile. Moreover, the characteristics of the patients with third tertile plasma BNP appear to be consistent with the characteristics of elderly heart failure patients. [30] On the other hand, there was no difference in motor FIM at admission between the first and third tertile groups. This is likely due to the patients’ inability to move immediately after hip fracture, irrespective of the presence of heart failure. Subsequently, however, as rehabilitation improved ADL, patients with high plasma BNP levels showed less improvement than those with low plasma BNP.

Increased left ventricular filling pressure is a fundamental hemodynamic abnormality in heart failure, and plasma BNP levels correlate well with left ventricular end-diastolic pressure and pulmonary capillary wedge pressure (PCWP) in patients with heart failure. [31] Recent studies also showed that during exercise, heart failure patients display higher PCWPs and pulmonary artery pressures, higher Borg perceived dyspnea scores, and increased ventilatory drive and respiratory rate than healthy controls. [32,33] Thus, exercise-induced altered hemodynamics, abnormal respiratory function, and dyspnea are characteristic of heart failure patients, and likely lead to reduced exercise endurance. Consequently, patients with hip fractures and heart failure defined by higher plasma BNP levels are unable to receive the same degree of rehabilitation as those without heart failure, which likely decreases ADL improvement.

We also divided patients into tertiles based on REs. We found that patients in whom REs was lower exhibited higher plasma BNP, older age, poorer handgrip strength, poorer nutrition, and poorer swallowing function–i.e., characteristics similar to those of patients in the third BNP tertile. In other words, REs appears to be lower in hip fracture patients with heart failure. That finding is consistent with our results showing that the effect of rehabilitation on ADL improvement was significantly blunted in the High BNP group as compared to the Low BNP group (Fig 4).

In addition to heart failure assessed based on plasma BNP levels, nutrition, age, swallowing function, handgrip strength, and gender were also related to REs in patients with hip fracture. Previously, Semel et al. [9] showed that younger age and female gender were significantly associated with length of stay efficiency, which was calculated as the FIM gain divided by the length of stay in patients with hip fracture. Inoue et al. [8] also reported that malnutrition status, as determined using MNA-SF, is a negative predictor of motor FIM gain in patients with hip fracture. Those findings are consistent with our present results, which is indicative of the validity of our patient cohort and the rehabilitation they were provided.

### Limitations

The present study has several limitations. First, it was a single-center investigation performed in a limited number of patients. Therefore, our findings should be confirmed in a multicenter study with a larger number of patients. Second, the etiology of heart failure was not investigated. Among the elderly in Japan, ischemic heart disease, valvular heart disease and hypertension are the three major heart failure etiologies [34]. Because PCWP rises during exercise in patients with ischemic [35], valvular [36], or hypertensive [37] heart disease and its elevation limits rehabilitation, even if the distribution of etiologies changes, we think it would have little impact on the our results. Third, we showed a slight difference in the relationship between RE and BNP levels between patients under the age of 83 and those over the age of 83. Patients in the age group older than 83 tended to have lower REs in the high BNP group, but did not reach statistical significance. Poor statistical power may be the cause due to the small number of patients. A slight difference in the relationship between REs and stage of heart failure was also observed between patients under the age of 83 and those over the age of 83. Patients under the age of 83 tended to have lower REs in the high BNP group, but did not reach statistical significance. A large number of patient studies will be needed in the future to confirm the results of this study. Fourth, we did not mention the cardiac rehabilitation. However, exercise-based cardiac rehabilitation is an established, safe and effective intervention to improve physical functional performance, muscle strength, and quality of life and is associated with a reduction in heart failure hospitalization in stable heart failure patients [38]. Therefore, following rehabilitation of hip fracture and acquisition of walking ability, it is desirable to connect these patients to home-based cardiac rehabilitation [39].

## Conclusions

In this study, we investigated whether heart failure affects the ADL improvement in patients after hip fracture. Using plasma BNP on admission as an index of heart failure and dividing the patients into tertiles based on plasma BNP levels, we found that heart failure negatively impacts improvement of ADL during rehabilitation of patients with hip fracture. This BNP based classification well matches heart failure stage classification according to the ACC/AHA heart failure guideline.

## List of abbreviations

ADL: activity of daily living
ANOVA: analysis of variance
ANCONA: analysis of covariance
BNP: B-type natriuretic peptide
CTR: cardio thoracic ratio
CRP: plasma C-reactive protein
FIM: functional independence measurement
FOIS: functional oral intake scale
GNRI: geriatric nutritional risk index
LOS: length of hospital stay
REs: rehabilitation effectiveness
